# Heterostructured ferromagnet–topological insulator with dual-phase magnetic properties†

**DOI:** 10.1039/c8ra00068a

**Published:** 2018-02-19

**Authors:** Shu-Jui Chang, Pei-Yu Chuang, Cheong-Wei Chong, Yu-Jung Chen, Jung-Chun Andrew Huang, Po-Wen Chen, Yuan-Chieh Tseng

**Affiliations:** Department of Materials Science & Engineering, National Chiao Tung University Hsinchu Taiwan yctseng21@mail.nctu.edu.tw; Department of Physics, National Cheng Kung University Tainan Taiwan; Advanced Optoelectronic Technology Center, National Cheng Kung University Tainan Taiwan; Taiwan Consortium of Emergent Crystalline Materials, Ministry of Science and Technology Taipei Taiwan; Division of Physics, Institute of Nuclear Energy Research Taoyuan Taiwan

## Abstract

The introduction of ferromagnetism at the surface of a topological insulator (TI) produces fascinating spin-charge phenomena. It has been assumed that these fascinating effects are associated with a homogeneous ferromagnetic (FM) layer possessing a single type of magnetic phase. However, we obtained phase separation within the FM layer of a Ni_80_Fe_20_/Bi_2_Se_3_ heterostructure. This phase separation was caused by the diffusion of Ni into Bi_2_Se_3_, forming a ternary magnetic phase of Ni:Bi_2_Se_3_. The inward diffusion of Ni led to the formation of an FeSe phase outward, transforming the original Ni_80_Fe_20_/Bi_2_Se_3_ into a sandwich structure comprising FeSe/Ni:Bi_2_Se_3_/Bi_2_Se_3_ with dual-phase magnetic characteristics similar to that driven by the proximity effect. Such a phenomenon might have been overlooked in previous studies with a strong focus on the proximity effect. X-ray magnetic spectroscopy revealed that FeSe and Ni:Bi_2_Se_3_ possess horizontal and perpendicular magnetic anisotropy, respectively. The overall magnetic order of the heterostructure can be easily tuned by adjusting the thickness of the Bi_2_Se_3_ as it compromises the magnetic orders of the two magnetic phases. This discovery is essential to the quantification of spin-charge phenomena in similar material combinations where the FM layer is composed of multiple elements.

## Abstract 2

In this study we visualized thermodynamically stable chalcogen compounds in the vicinity of a Py/Bi_2_Se_3_ interface with dual magnetic order due to a phase separation effect.

## Graphical abstract

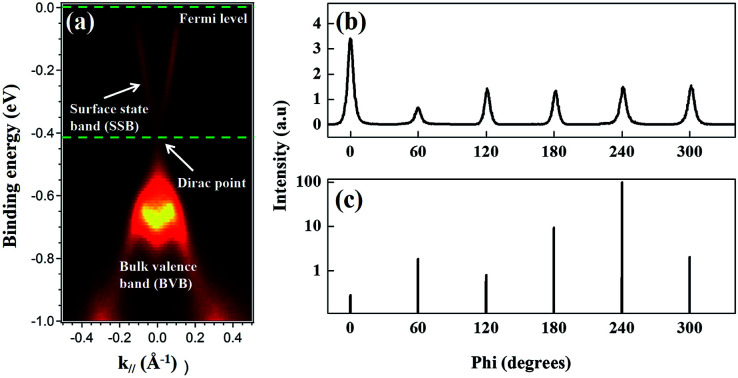

## Introduction

Heterostructures provide a versatile playground in which to manipulate the structural, electronic, optical and magnetic properties of thin films. New phases commonly emerge at the interface between dissimilar materials confined within a narrow region, resulting in exotic characteristics that differ significantly from those of bulk materials. The interior of a topological insulator (TI) resembles that of a conventional insulator; however, the surface state is conductive.^1–13^ Confining electrons to the surface state induces novel behaviour. For example, the momentum and spin of electrons in TIs are constrained to be perpendicular, due to strong spin–orbit interaction. Locking the spin momentum may enable the development of devices that would be impossible to produce using other semiconductor materials. A heterostructure comprising a TI and ferromagnet (FM) enables the conversion of charge-spin current with an extremely high efficiency.^14,15^ This opens many opportunities for spintronic applications, such as the development of devices with low power consumption and enhanced compatibility among devices controlled using integrated circuits. Optimizing charge-spin current conversion requires a sophisticated understanding of the structural, electronic and/or magnetic states, particularly at the FM/TI interface.

Ni_80_Fe_20_ permalloy (Py) is renowned for its permeability,^16,17^ low coercivity,^16,18,19^ near zero magnetostriction^20,21^ and ease of fabrication using inexpensive methods. Mellnik *et al.*^15^ recently demonstrated spin-transfer torque (STT) generated by a Py/Bi_2_Se_3_ heterostructure device. Tian *et al.*^22^ reported current-induced, persistent spin polarization in a Py/Bi_2_Te_2_Se material combination. Spin–orbit torque (SOT) has also been demonstrated in systems comprising CoFe and Bi_2_Se_3_.^23,24^ It is generally believed that these fascinating physical effects are due to the FM/TI heterostructure, based on the premise that the interface is atomically abrupt in cases where the FM layer comprises a pure single phase with one type of magnetic order. However, in this study, high-resolution transmission electron microscopy and synchrotron X-rays analysis revealed thermodynamically stable chalcogen compounds (F–Se and Ni–Se related phases) in the vicinity of a Py/Bi_2_Se_3_ interface with variable magnetic phase/order. These effects were observed despite the fact that the characteristics of Bi_2_Se_3_ TI remained intact. Our results revealed that an intermediate phase may unexpectedly emerge during the formation of the FM/TI; however, this field remains in its infancy. With the emergence of a new ground state, the striking effects associated with spin transfer/orbital torque and spin-momentum locking could be altered in an unpredictable manner as a result of the re-oriented magnetic order at the interface. This also raises concerns as to whether the fascinating behaviour mentioned above (*i.e.* STT and SOT) arises from a presumably perfect FM/TI interface or whether a third phase plays a secondary role to the proximity effect^25,26^ that has yet to be elucidated. This is a particularly thorny issue from the perspective of quantifying spin-charge phenomena, which is a crucial issue in spintronics. For example, our results indicate that some intermediate phases are highly sensitive to interfacial magnetic anisotropy. In this study, we sought to elucidate the development and behaviour of novel phases within FM/TI heterostructures to aid in the further development of heterostructures with predictable interfacial bonding.

## Experimental

### Method

Ag/Py/Bi_2_Se_3_ was sequentially deposited as a series of layers on a c-plane sapphire substrate. Bi_2_Se_3_ thin films were grown using a molecular beam epitaxy (MBE) system (AdNaNo Corp. mode, model MBE-9) in an ultra-high vacuum chamber with a base pressure of 2.75 × 10^−8^ Pa. Bi (99.99%) and Se (99.999%) precursors were evaporated using Knudsen cells to ensure control over the content settling on the substrate, with flux monitoring using a quartz crystal micro-balance. The growth temperature and Bi/Se flux ratio were respectively maintained at 290 °C and 1 : 15 under 1.33 × 10^−7^ Pa, resulting in growth rates of 0.2 to 0.3 QL per min.^27,28^ Prior to the deposition of the Py and Ag capping molecules, the sputtering chamber was evacuated to less than 6.66 × 10^−5^ Pa to avoid contamination. Py (2 nm) and Ag (2 nm) were deposited using radio frequency (RF) sputtering under 4.9 Pa at room temperature, resulting in growth rates of 0.48 and 3.24 nm min^−1^, respectively. MBE-grown Bi_2_Se_3_ was transferred directly into the sputter chamber without breaking the ultra-high vacuum. The thickness of the Bi_2_Se_3_ was varied (5 nm, 20 nm and 40 nm), and the samples were respectively denoted as Py/Bi_2_Se_3_-5 nm, Py/Bi_2_Se_3_-20 nm and Py/Bi_2_Se_3_-40 nm.

### Characterizations

Transmission electron microscopy was used to probe the microstructure and high-resolution imaging was used to obtain atomic-scale images. Spatially resolved energy dispersive X-ray (EDX) attached to TEM was used to characterize the compositional distribution across the interface. The epitaxial characteristics and crystallographic properties of the films were examined using synchrotron-based, high-resolution X-ray scattering with a photon energy of 8 keV. Synchrotron radiation photoelectron spectroscopy (SR-PES) was used to characterize the chemical structure, particularly on the sample surfaces. Angle-resolved photoemission spectroscopy (ARPES) was used (incident energy of 22 eV) to probe the Dirac point of the surface states of the Bi_2_Se_3_ films. The angular resolution was approximately 0.2°, which resulted in an energy resolution exceeding 12 meV. X-ray absorption spectra (XAS) and X-ray magnetic circular dichroism spectra (XMCD) were collected over Ni/Fe L_2_/L_3_-edges to provide element-specific, spin-dependent electronic information. All the XAS and XMCD data were respectively normalized to the post-edge jump and XAS integration. This was done to ensure reliable quantitative comparisons by regularizing the data with respect to any variations in absorber concentration and other aspects of the measurement. Magnetic-field and temperature-dependent magnetization measurements were conducted using a vibrating sample magnetometer (Quantum Design Versalab). The low-temperature magnetic properties (<50 K) were analyzed by a superconducting quantum interference device (SQUID) magnetometer.

## Results and discussion

**Fig. 1:**
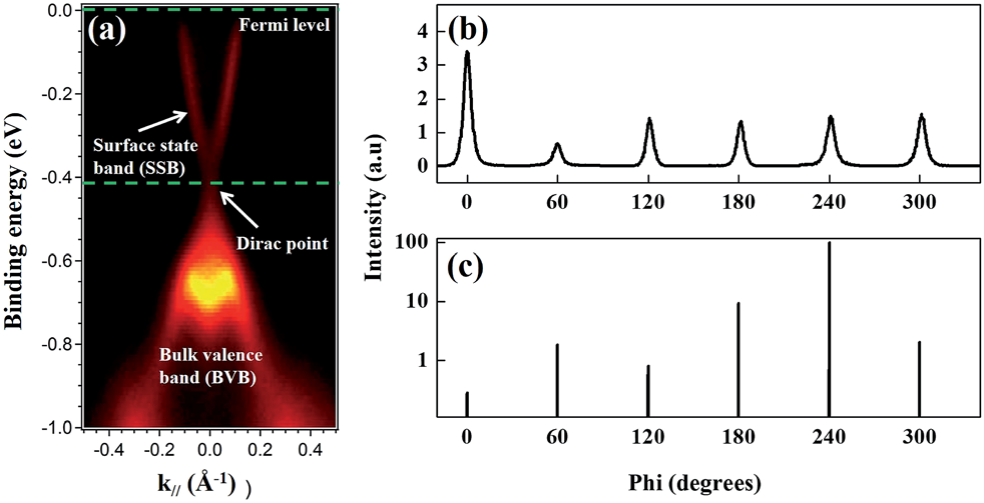
X-ray characterization of the electronic and structural properties. (a) ARPES spectra of Bi_2_Se_3_ film taken along the *Γ*–*Κ* direction, identifying details of the band structure (Fermi-level, surface state band, Dirac point and bulk valence band). X-ray azimuthal scans of the: (b) Bi_2_Se_3_ film and (c) sapphire substrate.

**Fig. 2:**
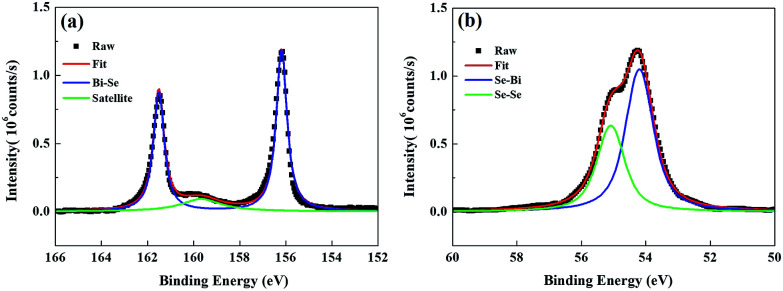
SR-PES spectra of the Bi_2_Se_3_ film. (a) Bi-4f and (b) Se-3d doublet peaks with curve fittings. The incident energy was fixed at 380 and 250 eV for Bi-4f and Se-3d, respectively.

Fig. 1(a) presents the ARPES band mapping of the Py/Bi_2_Se_3_-40 nm sample after removal of the Py capping layer (full disappearance of Ni and Fe signals in an element-resolved analyzer attached to ARPES) using Ar^+^ sputtering attached to the ARPES chamber. Therefore, the presented ARPES correspond to the pure bottom Bi_2_Se_3_. We observed two bands with a nearly linear dispersion at the *Γ* point, which formed a conical band representing the topological surface states (TSS) with the Dirac point at a binding energy of 0.4 eV. The binding energies exceeding 0.4 eV in the bulk valence band are characteristic of a TI and an indication that the MBE-grown Bi_2_Se_3_ possessed high crystallinity. Fig. 1(b) presents the azimuthal scan patterns at the {113} plane of the sapphire substrate (lower figure) and at the {015} plane of the Bi_2_Se_3_ film (upper figure) and it shows sharp diffraction peaks exhibiting the expected six-fold symmetry, where six-fold rotational peaks both for Bi_2_Se_3_ (015) and sapphire (113) with 60° can be identified, respectively. This confirms the in-plane orientation relationship between Bi_2_Se_3_ and the c-sapphire substrate. From a crystallographic perspective, this is an indication of epitaxial growth. Fig. 2(a) and (b) present the SR-PES spectra of Bi 4f and Se 3d in the as-grown Bi_2_Se_3_ without Py capping, where the X-ray incident energy was fixed at 380 and 250 eV, respectively. This approach was intended to acquire photoelectrons at a constant kinetic energy of 200 eV in order to optimize the surface sensitivity of pure Bi_2_Se_3_ by eliminating the influence from Py. The Bi/Se area ratio was estimated by curve-fitting as 1.49, indicative of a stoichiometric composition close to the ideal Bi_2_Se_3_ formula. Details of the curve-fitting are outlined in Fig. S-1.† The above results confirm the high quality of the Bi_2_Se_3_ grown by MBE.^27,28^

**Fig. 3:**
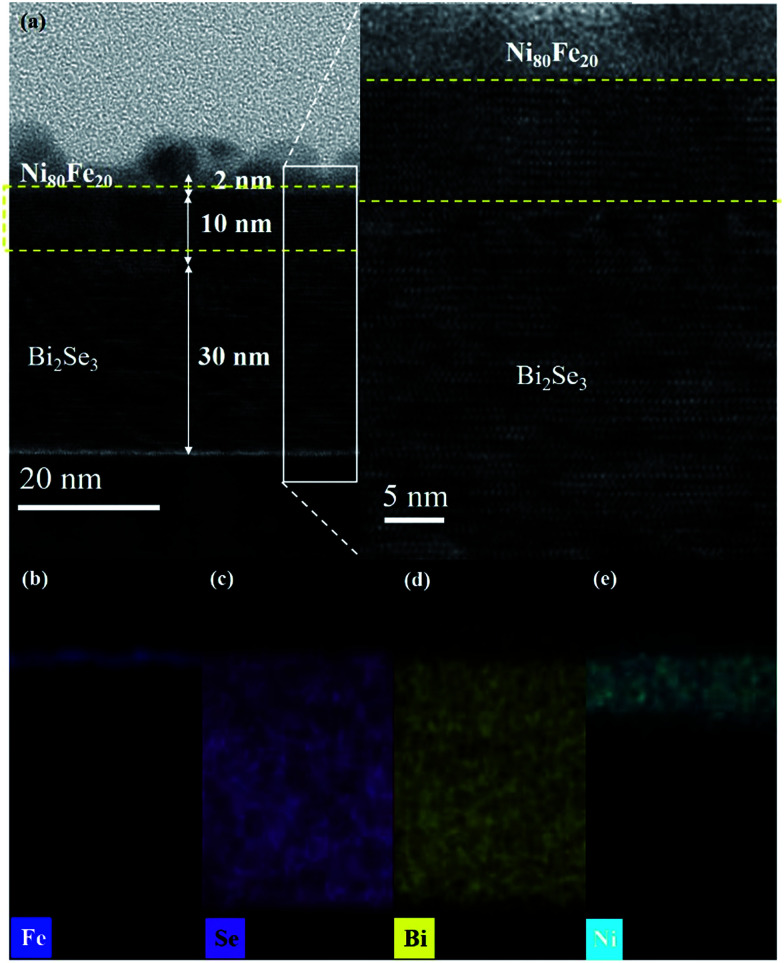
TEM characterization and EDX chemical mapping of the Py/Bi_2_Se_3_ heterostructure. (a) Cross-sectional TEM image of the heterostructure. EDX mapping of (b) Fe, (c) Se, (d) Bi and (e) Ni. The inset in (a) is an HRTEM image of the selected region of the heterostructure, in which the dashed lines identify the intermediate phases with crystallinity and lattice spacing different from those of amorphous Py (top) and epitaxial Bi_2_Se_3_ (bottom).

Fig. 3(a) presents a cross-sectional TEM image of the Ag/Py/Bi_2_Se_3_ heterostructure with a Bi_2_Se_3_ thickness of 40 nm. Fig. 3(b), (c), (d), (e) present spatial elemental-mapping of Fe, Ni, Bi and Se, respectively. Discontinuities in the Ag capping layer were unavoidable, due to the use of a focused ion beam during the TEM preparation process.^29,30^ An intermediate phase (thickness of ∼10 nm) was observed between the amorphous Py and epitaxial Bi_2_Se_3_. Spatial elemental-mapping revealed a homogeneous phase of Bi_2_Se_3_, with Bi and Se uniformly populating the Bi_2_Se_3_ layer. However, we observed that the Ni and Fe were phase-separated, with Fe preferentially remaining in the upper region and Ni penetrating into the Bi_2_Se_3_. The relatively rapid self-diffusion of Ni (exceeding that of Fe)^31,32^ enhanced diffusion towards the inner layer, which resulted in a new phase with Bi_2_Se_3_. This phase separation was responsible for the intermediate phase observed in the TEM images. We also obtained this intermediate phase in the other two samples (Py/Bi_2_Se_3_-5 nm and Py/Bi_2_Se_3_-20 nm), with detailed characterizations (TEM and EDX) given in Fig. S-2.† Note that the Py film was deposited using a high-quality, single-phase Ni_80_Fe_20_ target at a low deposition rate (0.48 nm min^−1^). The resulting films were also free from thermal-induced inter-diffusion, as annealing was not performed. In Fig. 3(c), the overlapping region between Ni and Bi_2_Se_3_ in the Py/Bi_2_Se_3_-40 nm sample is approximately 10 nm, which is far thicker than the Py layer (2 nm). The Ni:Bi_2_Se_3_ overlapping regions of the Py/Bi_2_Se_3_-5 nm and Py/Bi_2_Se_3_-20 nm samples were also obtained. These findings help to rule out the possibility that the phase separation was due to the extreme thinness of the Py layer. Instead, phase separation appears to be intrinsic to the Py when deposited on Bi_2_Se_3_. It should be noted that in Fig. 3(d), Se reached the upper Fe layer by diffusing across the Ni–Se overlapping region, whereas Bi remained within the Bi_2_Se_3_ layer. This implies that Se is more chemically active than Bi, and therefore plays a more important role in phase separation. The formation of FeSe and Ni:Bi_2_Se_3_ phases atop the Bi_2_Se_3_ layer was confirmed by the XANES results, as discussed later. Bi_2_Se_3_ is a layer-by-layer (LbL) structure with ionic-covalent bonded quintuple (QL) slabs formed by a periodic arrangement of layers aligned perpendicularly along the *z*-direction with weak van der Waals forces. Thus, there is a van der Waals gap (∼0.41 nm) between each QL.^33,34^ Ni can either intercalate into the van der Waals gaps between adjacent QLs, or occupy the interstitial sites within QL. This is because Ni has an elemental radius (125 pm) smaller than the van der Waals gap, which enables it to occupy interstitial sites.^34–36^ The Pauling electronegativity values of Ni and Fe are 1.91 and 1.83, respectively. Both of these values are lower than that that of Bi (2.02).

**Fig. 4:**
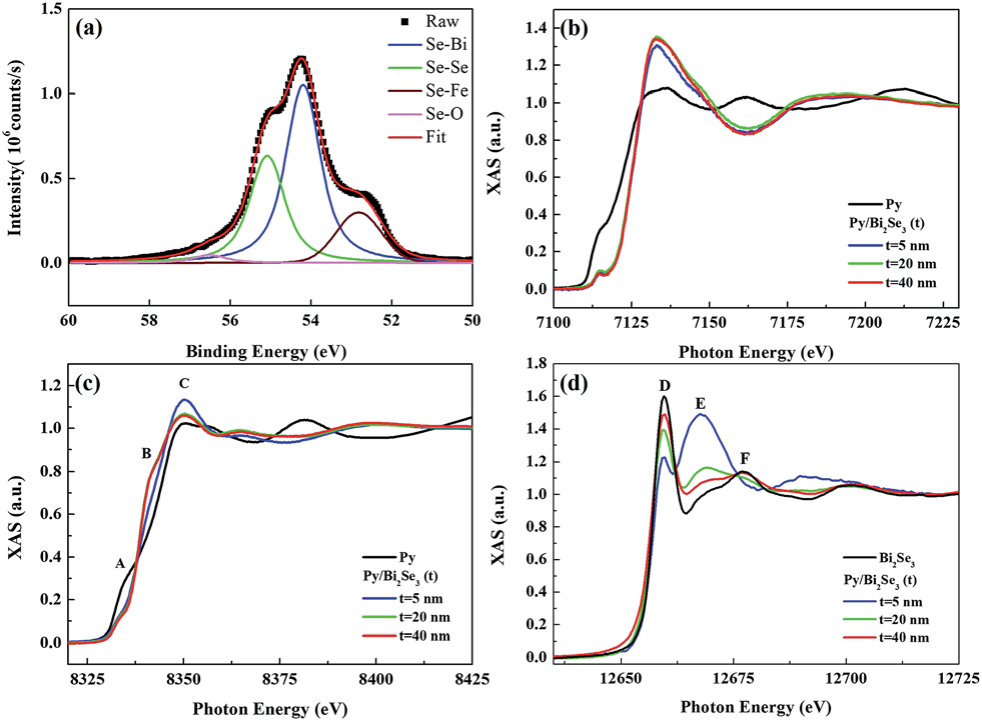
SR-PES and XANES of the Py/Bi_2_Se_3_ heterostructure. (a) SR-PES Se 3d spectrum of Py/Bi_2_Se_3_-40 nm heterostructure after Ar ion etching for 8 min at 2 keV, showing curve-fitting for Se–Bi, Se–Se, Se–Fe, Se–O bonding; (b) Fe K-edge, (c) Ni K-edge, and (d) Se K-edge XANES spectra with Bi_2_Se_3_ thickness-dependence.

The two above-mentioned effects are possibly responsible for the formation of Ni:Bi_2_Se_3_ (ref. 37–39) and FeSe compounds^40,41^ as considering the XANES results. The inset of Fig. 3(a) presents a high-resolution image of the interface. The as-deposited Py exhibits an amorphous microstructure. We probed the chemical state and local coordination of the intermediate phase using SR-PES and XANES, respectively. Fig. 4(a) presents the SR-PES of Se 3d obtained from the Py/Bi_2_Se_3_-40 nm sample. This is an indication of the extreme sensitivity in the uppermost region of the film after the capping of Bi_2_Se_3_ with Py. Compared to Fig. 2(b), we can see an additional shoulder in the Se 3d spectra at a binding energy of ∼52.8 eV, which corresponds to Fe–Se bonding. It appears that the capping of Bi_2_Se_3_ with Py resulted in a new phase of FeSe. Fig. 4(b) and (c) compare the K-edge XANES spectra of Fe and Ni for the thickness-dependent Py/Bi_2_Se_3_ and pure Py. The Fe XANES results for pure Py are identical to those for metallic Fe,^42,43^ whereas the Fe XANES line-shape from Py/Bi_2_Se_3_ is similar to that of FeSe, with the p-like symmetry indicating strong covalent bonding.^44,45^ The Se XPS and Fe XANES results suggest that the phase-separated Fe formed a FeSe phase in the uppermost region of the film. Ni was also electronically modified by Bi_2_Se_3_; however, this resulted in a XANES line-shape different from that of Py. The reformed Ni XANES line-shape suggests the formation of Ni dichalcogenides (NiSe_*x*_).^46,47^ We observed three major structures in the Ni XANES results. The pre-edge feature at ∼8333 eV (peak A) corresponds to an electronic transition to empty e_g_ states. The second structure was observed at ∼8339 eV (peak B), which can be attributed to a transition to the Ni 4sp band. The third structure at ∼8349 eV (white-line peak, peak C) is related to a p-like symmetry state, which is typically sensitive to Ni's d orbital mixing with Se 4p bands.^47,48^ Fig. S-3† outlines the differential Ni XANES results used to indicate such a delicate transition.

Fig. 4(d) presents the Se K-edge XANES results of all the samples in this investigation, together with the Se K-edge XANES of pure Bi_2_Se_3_ without Py capping. The XANES line-shape suggests that the compound features an Se^2−^ electronic state, as identified by peaks D, E and F.^40,41^ Peak D (∼12 659 eV) is associated with hybridized Se p-TM (TM = Ni and Fe) d states. Peak E (∼12 668 eV) is associated with the e_g_ antibonding state associated with the Se site. This state is related to the local structure of the Se and is a dominant feature of FeSe compounds.^40,41^ Peak F (∼12 677 eV) refers to multiple scattering from the symmetrical Se 4sp states in the coordination surroundings. It is more evident in Bi_2_Se_3_ than in TM selenides.^49,50^ The oscillation patterns (except for peak D) of pure Bi_2_Se_3_ were more distinct than those of Py/Bi_2_Se_3_. This was expected, due to the fact that the absorption edge exhibits a strong dependence on the peak position with respect to the changes in TM hybridization with Se. This also means that from the perspective of Se, the electronic and atomic coupling was altered by the introduction of Py. Given that FeSe is independent of Bi_2_Se_3_ thickness (Fig. 4(b)), an increase in the thickness of Bi_2_Se_3_ empties the Se p state, as reflected by the increase in the intensity of peak D. This reflects an increase in the contribution of Bi to bonding in these structures.^49,51^ The multiple scattering of peak E presents a slight shift towards higher energies, mainly due to changes in the local geometry around the Se atoms.^49,52^ This points to a stronger Bi–Se hybridization. Peak E appeared prominent in the thinnest Bi_2_Se_3_ sample (Py/Bi_2_Se_3_-5 nm). The fact that this peak is a dominant feature of FeSe^40,41^ means that FeSe is more dominant in the Py/Bi_2_Se_3_-5 nm sample due to the thinnest (*i.e.* weakest) Bi_2_Se_3_ layer. Increasing the thickness of the Bi_2_Se_3_ destabilized the Bi_2_Se_3_ phase (peak F) at the expense of FeSe (peak E).

Our TEM and XANES results confirm that all of the samples presented a FeSe/Ni:Bi_2_Se_3_/Bi_2_Se_3_ sandwiched structure. In all of the samples, the thickness of the FeSe phase was ∼2 nm. The thickness of the Ni:Bi_2_Se_3_ phase was as follows: Py/Bi_2_Se_3_-5 nm (1.5 nm), Py/Bi_2_Se_3_-20 nm (8 nm) and Py/Bi_2_Se_3_-40 nm (10 nm). When starting with the same Py thickness (2 nm), we found that an increase in Bi_2_Se_3_ made the Ni:Bi_2_Se_3_ phase more robust, indicating that Bi_2_Se_3_ has a strong tendency to form the Ni:Bi_2_Se_3_ phase. In other words, Ni:Bi_2_Se_3_ behaves like a second ferromagnetic phase^53,54^ (after FeSe). The presence of two intermediate phases can produce a magnetic order different from that of pure Py; however, this minimizes the likelihood of changes in the topological properties of Bi_2_Se_3_. This may explain why the issue of phase separation in the FM layer was disregarded in previous studies on Py/Bi_2_Se_3_ and CoFe/Bi_2_Te_2_Se.^15,22–24^

**Fig. 5:**
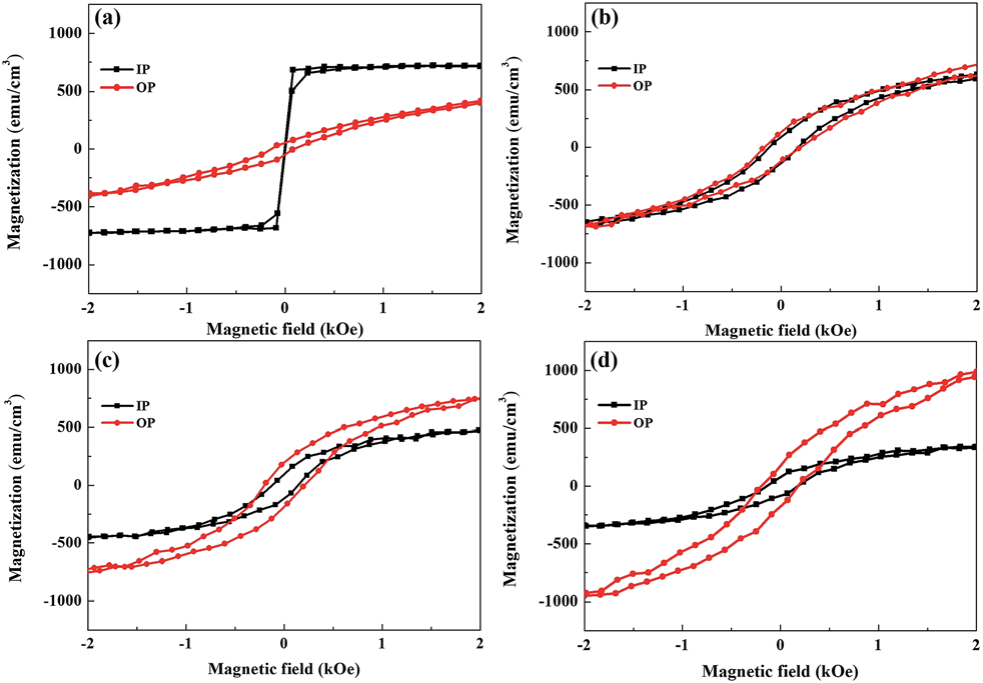
Room temperature magnetic properties. *M*–*H* curves with Bi_2_Se_3_ thickness-dependence measured at 300 K, along the in-plane (IP) and out-of-plane (OP) directions: (a) pure Py, (b) Py/Bi_2_Se_3_-5 nm, (c) Py/Bi_2_Se_3_-20 nm and (d) Py/Bi_2_Se_3_-40 nm. All the *M*–*H* curves were normalized to the sample area to ensure a reasonable quantitative comparison.

**Fig. 6:**
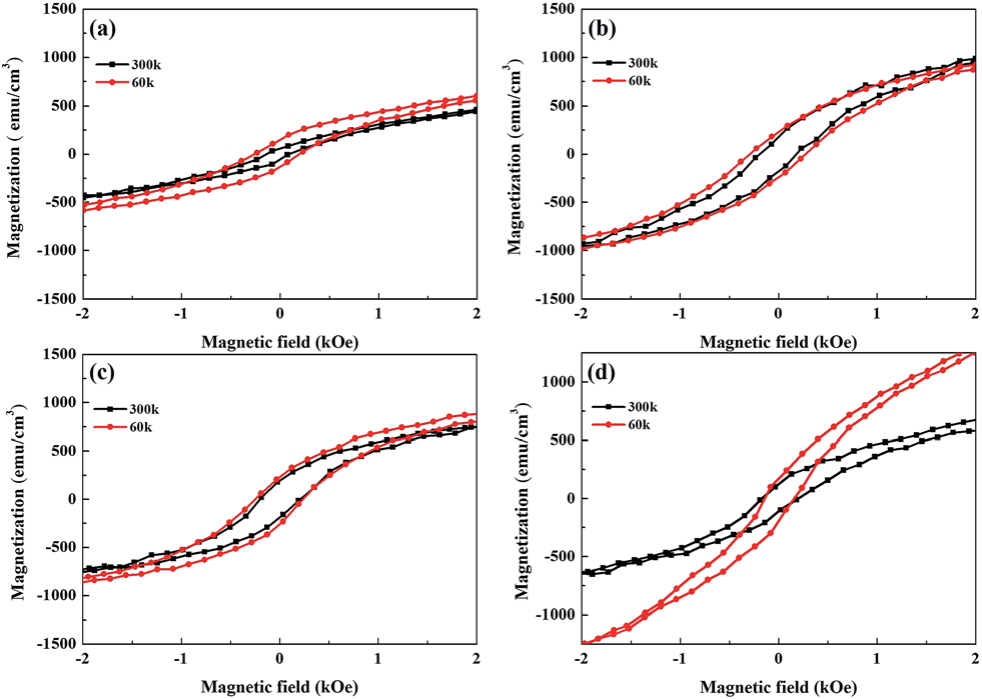
Temperature-dependent magnetic properties. Out-of-plane *M*–*H* curves with Bi_2_Se_3_ thickness-dependence measured at 60 and 300 K, for: (a) pure Py, (b) Py/Bi_2_Se_3_-5 nm, (c) Py/Bi_2_Se_3_-20 nm and (d) Py/Bi_2_Se_3_-40 nm. All the *M*–*H* curves were normalized to the sample area to ensure a reasonable quantitative comparison.

In the following, we examine the magnetic hysteresis (*M*–*H*) loops in order to shed light on the issue of the magnetic order. All the *M*–*H* data were normalized to the volume of the corresponding sample to ensure reasonable quantitative comparisons. Fig. 5(a) presents the in-plane and out-of-plane *M*–*H* of pure Py at room temperature. The pure Py exhibits strong in-plane anisotropy (IMA), as described in previous studies.^18,19,55,56^Fig. 5(b), (c) and (d) present the in-plane and out-of-plane *M*–*H* for the Py/Bi_2_Se_3_-5 nm, Py/Bi_2_Se_3_-20 nm and Py/Bi_2_Se_3_-40 nm samples, respectively. Interestingly, in the presence of Bi_2_Se_3_, Py loses IMA, and its saturation magnetization (*M*_s_) is heavily suppressed. This implies that the formation of FeSe and Ni:Bi_2_Se_3_ weaken the magnetic order of the original Py, and there exists a magnetic re-orientation as the fraction of FeSe/Ni:Bi_2_Se_3_ changes. This was supported by the in-plane and out-of-plane remanence ratios *M*_0_/*M*_sat_, as shown in Fig. S-4,† wherein an increase in the thickness of Bi_2_Se_3_ caused a switch from IMA to PMA. This could be due to the proximity effect or interface-induced changes in magnetization. Previous studies found that by breaking the time-reversal symmetry of TI using magnetic dopants^57^ or FM capping,^26^ strong spin–orbit coupling could be used to modify magnetic anisotropy, leading to an out-of-plane magnetic moment in TI. Fig. 6(a), (b), (c) and (d) present the temperature-dependent (60 and 300 K) out-of-plane *M*–*H* for pure the Py, Py/Bi_2_Se_3_-5 nm, Py/Bi_2_Se_3_-20 nm and Py/Bi_2_Se_3_-40 nm samples, respectively, where the *M*–*H* data were collected by field-cooling the samples with a magnetic field of 2 Tesla. *H*_c_ enhancement and exchange bias were not obtained at low temperature. This rules out the possibility of an antiferromagnetic (AFM)^25^ or interface canting^26^ contribution to the IMA-PMA switch through the proximity effect. Besides, from the temperature-dependent magnetization data (Fig. S-5†), we found that the out-of-plane *M*_s_ was significantly promoted over the in-plane *M*_s_ with the increase of Bi_2_Se_3_ thickness over a broad temperature range. This was somehow unexpected from the proximity effect, because the proximity effect typically diminishes at high temperature. All these facts suggest that there exists another factor (*i.e.* a phase separation effect) additional to the proximity effect that is likely responsible for the IMA-PMA switch.

**Fig. 7:**
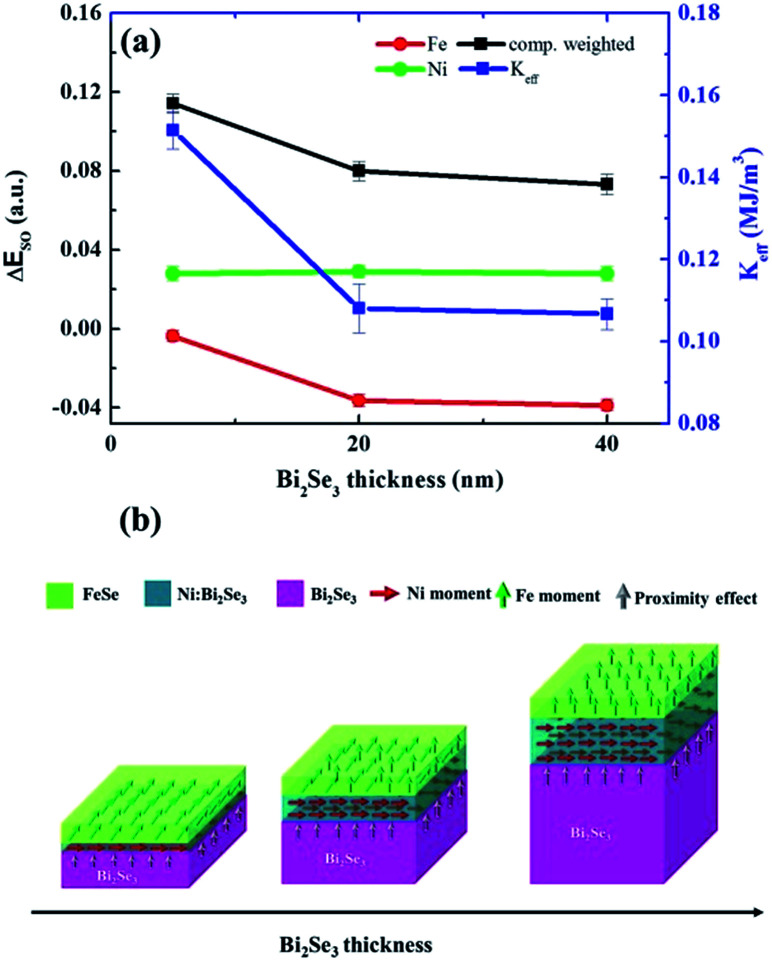
Spin–orbit energy calculations. (a) Dependence of Fe-, Ni- and composition-weighted-spin–orbit energy (Δ*E*_SO_) (unit on left *y*-axis) on Bi_2_Se_3_ thickness. *K*_eff_ (unit on right *y*-axis) *versus* Bi_2_Se_3_ thickness superimposed for comparison. (b) Schematic illustration showing the evolution of the interfacial magnetic orders of FeSe and Ni:Bi_2_Se_3_ phases in response to changes in the thickness of Bi_2_Se_3_. Green and red arrows refer to Fe and Ni moments, respectively. The proximity effect (golden arrows) is included.

To understand the moment alignment with respect to the dual magnetic phase from an atomic viewpoint, we used Fe/Ni XMCD along the in-plane and out-of-plane directions to calculate the spin–orbit energy (Δ*E*_SO_) according to the sum rule.^58,59^ The obtained Fe/Ni XMCD signals confirmed that FeSe and Ni:Bi_2_Se_3_ have a ferromagnetic and ferromagnetic–insulator phase, respectively. Details of these calculations are presented in Fig. S-6.† Magnetocrystalline anisotropy (MCA) in magnetic thin films is based on symmetry breaking at the interface, where the MCA energy corresponds to the energetic difference in spin orientation. According to the Bruno model,^60^ Δ*E*_SO_ is a physical quantity correlating the MCA energy and the orbital moment of the probed element, referring to the magnetic alignment along the out-of-plane (negative Δ*E*_SO_) or in-plane (positive Δ*E*_SO_) direction, depending on the sign of Δ*E*_SO_. Fig. 7(a) compares Δ*E*_SO_ and the effective MCA constant (*K*_eff_) in terms of Bi_2_Se_3_ thickness. *K*_eff_ is calculated from *M*–*H*. Interestingly, the Δ*E*_SO_ of Fe is weakened by an increase in Bi_2_Se_3_ thickness, whereas the Δ*E*_SO_ of Ni is independent of Bi_2_Se_3_ thickness. This provides straightforward evidence that the FeSe phase determines the changes in the magnetic anisotropy observed in thickness-dependent *M*–*H* (Fig. 5). The fact that the Δ*E*_SO_ of Fe is negative in all the samples means that the FeSe phase is of a weak PMA in nature; however, this is increased by an increase in the thickness of Bi_2_Se_3_. Conversely, the positive Δ*E*_SO_ of Ni is indicative of the IMA of Ni:Bi_2_Se_3_, which is independent of Bi_2_Se_3_ thickness. It is worth noting that the two magnetic phases have opposite magnetic orders. We calculated the composition-weighted Δ*E*_SO_ (80% Ni and 20% Fe) and superimposed this in Fig. 7(a). The composition-weighted Δ*E*_SO_ refers to the overall anisotropy compromised by FeSe and Ni:Bi_2_Se_3_ with their quantities. The trend of weighted Δ*E*_SO_ coincides with that of *K*_eff_ in terms of Bi_2_Se_3_ thickness. This presents a consistent picture from the atomic (*i.e.* Δ*E*_SO_) and macroscopic (*i.e. K*_eff_) perspectives. This, in turn, only strengthens the fact that phase separation causes a re-orientation in the magnetic order and helps to explain how FeSe and Ni:Bi_2_Se_3_ interact. In Fig. 7(b), we illustrate the evolution of the two types of magnetic order with changes in the thickness of Bi_2_Se_3_. Given a Bi_2_Se_3_ layer 5 nm in thickness, FeSe exhibited a very weak PMA. Increasing the thickness of Bi_2_Se_3_ strengthens the PMA of FeSe by lowering Δ*E*_SO_, reaching the highest value when Bi_2_Se_3_ was 40 nm thick. Nevertheless, the IMA nature of Ni:Bi_2_Se_3_ persisted throughout the entire range of thicknesses. It appears that the magnetic coupling between the two magnetic phases determines the overall magnetic order of the heterostructure. However, we believe that the proximity effect (golden arrows in Fig. 7(b)) cannot be ignored in the anisotropy-switching mechanism in consideration of the enhanced perpendicular magnetization at low temperature (Fig. 6 and S-5†), but its influence on the overall magnetic order is secondary to the phase separation effect in this case.

## Conclusions

TIs are known for their unique surface state, wherein electrons are extremely mobile but the bulk material is not conductive. The fact that electrons carry spin means that TIs might make spintronic components feasible. Thus, the integration of TIs with FM materials is attracting considerable attention in the scientific community. Despite an awareness of the importance of the FM/TI heterostructure, the homogeneity of the interface still has much to be understood. This raises the question of whether interface-driven phenomena are only associated with the proximity effect based on a single, homogeneous FM phase with one type of magnetic phase, or whether they arise from a dual magnetic phase caused by inter-diffusion, in which the macroscopically observed magnetic order is a compromised phenomenon. This work demonstrates the existence of phase separation in the Py/Bi_2_Se_3_ heterostructure. It is possible that this phenomenon exists in other similar material combinations but has been disregarded due to its negligible influence on TI properties. This work also revealed strong coupling between the two magnetic phases. Refinement of the TI phase increases the versatility of the heterostructure from the perspective of magnetic order. We found that controlling the magnetic order relies on manipulation of the separate FeSe and Ni:Bi_2_Se_3_ phases *via* magnetic coupling. Despite a global effort to elucidate the unique properties of TIs, this is the first work to discover these unexpected behaviours beyond the proximity effect at the FM/TI interface.

## Conflicts of interest

There are no conflicts to declare.

## Supplementary Material

RA-008-C8RA00068A-s001
