# Supplementary material for: Heterostructured ferromagnet–topological insulator with dual-phase magnetic properties
Source: RSC Adv. 2018 Feb 19;8(14):7785–91. doi: 10.1039/c8ra00068a (PMC9078499; doi:10.1039/c8ra00068a)
Supplement: RA-008-C8RA00068A-s001 [file RA-008-C8RA00068A-s001.pdf]

Electronic Supplementary Material (ESI) for RSC Advances.
This journal is © The Royal Society of Chemistry 2018

Supplementary Information

Heterostructured Ferromagnet-Topological Insulator with Dual-phase Magnetic Properties

Shu-Jui Chang [1], Pei-Yu Chuang[2] , Cheong-Wei Chong[3], Yu-Jung Chen[4], Jung-Chun-Andrew Huang[2,3,4] , Po-Wen Chen, and Yuan-Chieh Tseng[1]*

(1) Dept. Materials Science & Engineering, National Chiao Tung University, Hsinchu, Taiwan.
(2) Dept. Physics, National Cheng Kung University, Tainan, Taiwan.
(3) Advanced Optoelectronic Technology Center, National Cheng Kung University, Tainan, Taiwan.
(4) Taiwan Consortium of Emergent Crystalline Materials, Ministry of Science and Technology, Taipei, Taiwan.
(5) Division of Physics, Institute of Nuclear Energy Research, Taoyuan, Taiwan.
E-mail address:yctseng21@mail.nctu.edu.tw.

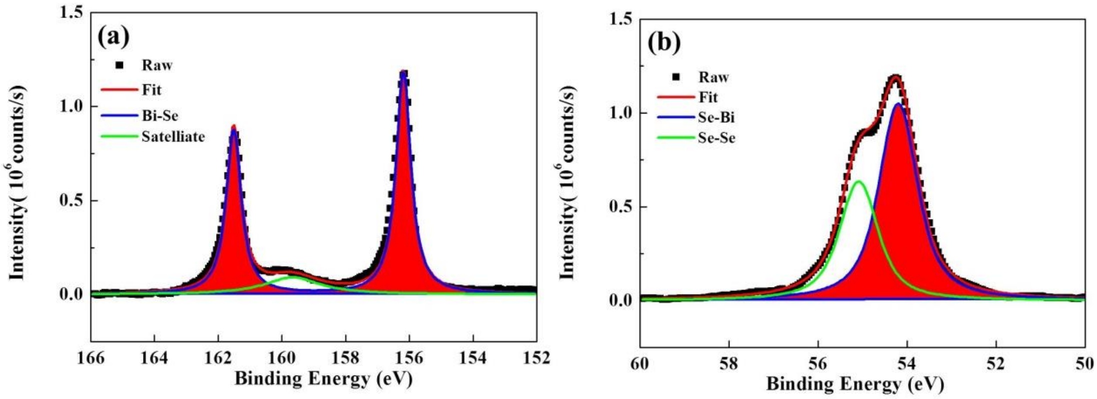

Fig. S-1 SR-PES spectra of (a) Bi-4*f* and (b) Se-3*d* where highlighted areas are used for Bi/Se ratio calculation, with the use of following equation:

$$n = \frac{I}{S}$$

where

$$S = f\sigma\theta y\lambda AT$$

where f is the Photon flux of the x-ray, σ is the photoionization cross-section of peak of element, θ is the photoelectron emission angle, y is the photoelectron yield, λ is the inelastic electron mean-free path, A is analysis area and T is a analyzer transmission function. Here, we define S is the sensitivity factor of core level of element.

Hence, n is dependent on the peak area and sensitivity factor.

$$\frac{n_1}{n_2} = \frac{(I_1 / S_1)}{(I_2 / S_2)} \quad (1)$$

The calculated Bi/Se ratio is 1.49 very close to the ideal stoichiometry of Bi_2Se_3 formula.

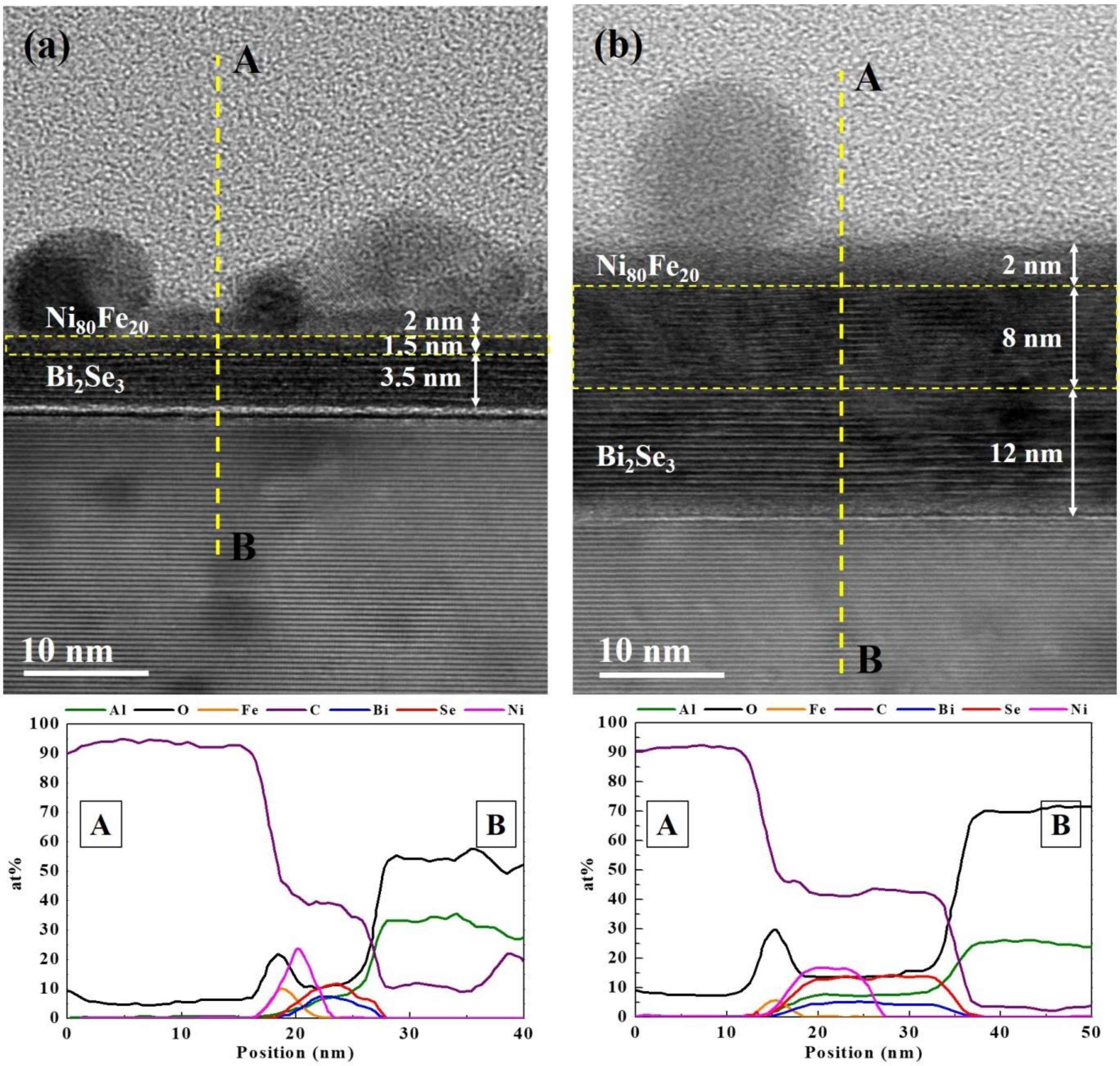

Fig. S-2 Cross-sectional TEM images of (a) Py/Bi_2Se_3-5nm and (b) Py/Bi_2Se_3-20 nm samples. EDX analyses are shown below the corresponding TEM images, where EDX line-scanning was performed along the yellow dashed line which starts from A and ends at B. EDX shows Ni diffusion into Bi_2Se_3 and it formed an Ni-Se overlapping region.

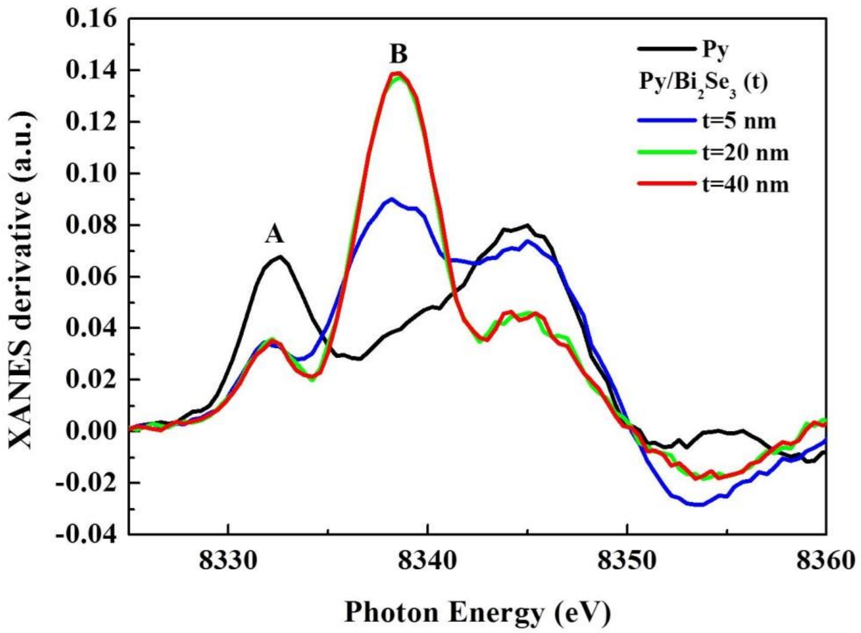

Fig. S-3 Differentiation of Ni *K*-edge XANES for pure Py (black), Py/Bi_2Se_3-5nm, Py/Bi_2Se_3-20nm and Py/Bi_2Se_3-40nm samples. The edge characteristics can be decomposed into feature A and B upon differentiation. Feature A is allowed by quadrupole selection rules. The weak intensity corresponds to a quadrupole 1*s*→3*d* dipole-forbidden transitions as a characteristic of chemical environment in Ni valency in which the intensity strongly depends upon the coordination symmetry. Feature A becomes less intense in Ni:Bi_2Se_3 as compared to Py, suggesting a local-structural change to centro-symmetry in coordination [1-3]. Feature B is attributed to the 1*s*→4*p* transition, which arises from a chalcogen-based charge-transfer and indicates the presence of strong Ni-Se covalent bonds. Feature B is intensified with the increase of Bi_2Se_3 thickness, implying a more stabilized phase of Ni:Bi_2Se_3. On the contrary, feature B is heavily suppressed in pure Py, which is a straightforward indicator to the absence of Ni:Bi_2Se_3.

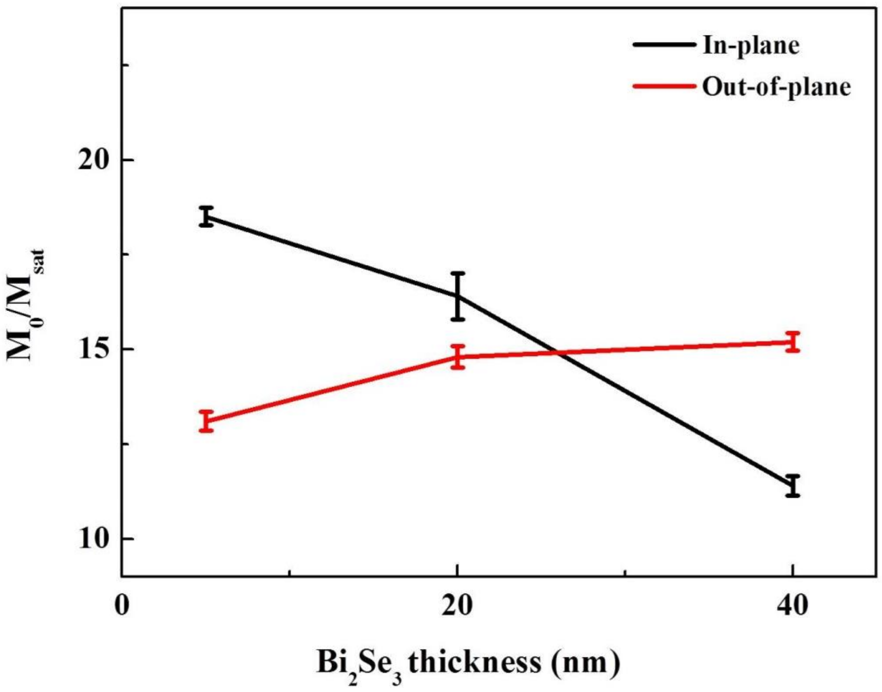

Fig. S-4 The dependence of remanence ratios (M_o/M_{sat}) on Bi_2Se_3 thickness, for in-plane and out-of-plane directions. M_o/M_{sat} was estimated from M-H curves.

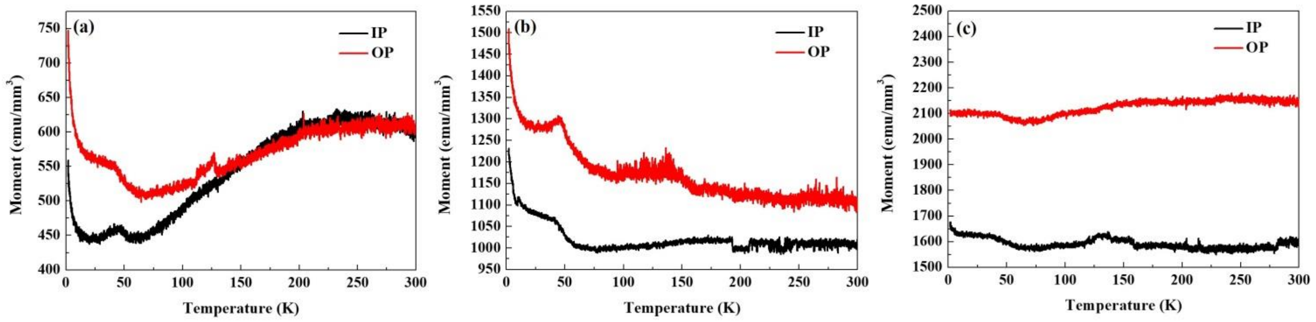

Fig. S-5 The temperature dependence of magnetization of (a) Py/Bi_2Se_3-5 nm, (b) Py/Bi_2Se_3-20 nm, (c) Py/Bi_2Se_3-40 nm, for in-plane (black) and out-of-plane (red) directions.

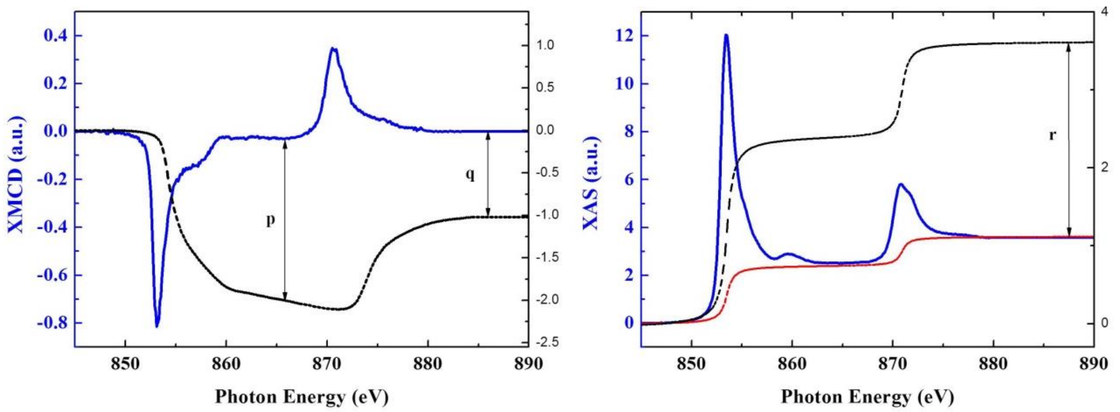

Fig. S-6 (left) Ni-XMCD and (right) Ni-XAS integrations applied in sum-rules analysis for the calculation of Ni's ΔE_{SO}. The orbital (m_{orb}) and spin (m_{spin}) moments are obtained by following equations:

$$m_{orb} = -4q(10-n_{3d})/3r \quad (1)$$

$$m_{spin} = -(6p-4q)(10-n_{3d})/r \quad (2)$$

Where

$$q = \int_{L3+L2} (\mu_+ - \mu_-)\,\mathrm{d}\varpi$$

$$r = \int_{L3+L2} (\mu_+ + \mu_-)\,\mathrm{d}\varpi$$

$$p = \int_{L3} (\mu_+ - \mu_-)\,\mathrm{d}\varpi$$

where μ_+ / μ_- is the x-ray absorption intensity with left/right circular polarization; n_{3d} is the number of 3*d* electron per cation and the ***p***, ***q*** and ***r*** are integrated values obtained from XMCD and XAS.

The calculated Ni m_{orb} are 0.02, 0.018 and 0.01 for Py/Bi_2Se_3-5 nm, Py/Bi_2Se_3-20 nm, and Py/Bi_2Se_3-40 nm, respectively, along in-plane direction, which is denoted $m_{orb}^{\parallel}$

Ni m_{orb} are 0.07, 0.07, 0.06 for Py/Bi_2Se_3-5 nm, Py/Bi_2Se_3-20 nm, and Py/Bi_2Se_3-40 nm, respectively, for out-of-plane direction, which is denoted $m_{orb}^{\perp}$.

In a simple model of Bruno [4][5], a relation between m_{orb} and ΔE_{SO} is derived as:

$$E_{SO} = -\frac{1}{2}\frac{\xi}{2\mu_B}[m_{orb}^{\perp} + (m_{orb}^{\parallel} - m_{orb}^{\perp})\sin^2\theta]$$

The energy difference for the magnetization aligned along out-of-plane and in-plane directions is:

$$\Delta E_{SO} = E_{SO}(\theta = 0°) - E_{SO}(\theta = 90°)$$

$$\Delta E_{SO} = -\frac{1}{2}\frac{\xi}{2\mu_B}[m_{orb}^{\perp} - m_{orb}^{\parallel}]$$

where ξ is spin-orbit coupling parameter for 3*d* electron and the values of $m_{orb}^{\perp}$ and $m_{orb}^{\parallel}$ are obtained by

sum-rules [6] along the out-of- and in-plane directions, respectively.

The calculated $m_{orb}^{\parallel} - m_{orb}^{\perp}$ values are 0.02795, 0.02907 and 0.02795, for Py/Bi_2Se_3-5 nm, Py/Bi_2Se_3-20 nm, and Py/Bi_2Se_3-40 nm, respectively.

The same method was used in the calculation of Fe's ΔE_{SO}.

References

[1] F. E. Huggins, K. C. Galbreath, K. E. Eylands, L. L. Van Loon, J. A. Olson, E. J. Zillioux, S. G. Ward, P. A. Lynch, and P. Chu, Determination of nickel species in stack emissions from eight residual oil-fired utility steam-generating units, *Environ. Sci. Technol.* **45**, 6188 (2011).

[2] A. Kisiel, P. Zajdel, P. M. Lee, E. Burattini, and W. Giriat, XANES study of K edges of Fe, Co, Ni, and Se in transition metal selenides. Experiment and comparison with LMTO numerical calculations, *J. Alloys Compd.* **286**, 61 (1999).

[3] S. A. Yao, V. Martin-Diaconescu, I. Infante, K. M. Lancaster, A. W. Gotz, S. DeBeer, and J. F. Berry, Electronic structure of Ni_2E complexes (E = S, Se, Te) and a global analysis of M_2E_2 compounds: a case for quantized E_2^{n-} oxidation levels with n = 2, 3, or 4, *J. Am. Chem. Soc.* **137**, 4993 (2015).

[4] D. Weller, Y. Wu, J. Stöhr, M. G. Samant, B. D. Hermsmeier, and C. Chappert, Orbital magnetic moments of Co in multilayers with perpendicular magnetic anisotropy, *Phys. Rev. B* **49**, 12888 (1994).

[5] J. Stöhr, Exploring the microscopic origin of magnetic anisotropies with X-ray magnetic circular dichroism (XMCD) spectroscopy, *J. Magn. Magn. Mater.* **200**, 470 (1999).

[6] P. Bruno, Tight-binding approach to the orbital magnetic moment and magnetocrystalline anisotropy of transition-metal monolayers, *Phys. Rev. B* **39**, 865 (1989).
